# Evaluation of Ficolin-3 deficiency as a risk factor in the development of rheumatic heart disease

**DOI:** 10.1186/s13104-025-07251-x

**Published:** 2025-05-08

**Authors:** Zahra Parker, M Taariq Salie, Kélin Engel, Liesl J Zühlke, Mark E Engel, Timothy F Spracklen

**Affiliations:** 1 Cape Heart Institute, Department of Medicine, Faculty of Health Sciences, University of Cape Town, Cape Town, South Africa; 2 South African Medical Research Council, Cape Town, South Africa; 3 Department of Paediatrics and Child Health, Faculty of Health Sciences, University of Cape Town, Cape Town, South Africa; 4 Cochrane South Africa, South African Medical Research Council, Cape Town, South Africa

**Keywords:** Rheumatic heart disease, Ficolin, Genetics, Africa

## Abstract

**Objective:**

Ficolin-3 is a crucial protein for the activation of the complement system. Previous work has indicated this protein may play a role in the pathogenesis of rheumatic heart disease (RHD), and it has been hypothesised that ficolin-3 has potential as a biomarker for early identification of patients with suspected RHD. This study investigated *FCN3* gene polymorphisms rs532781899 (c.349del) and rs4494157 (c.658 + 250 C > A) and ficolin-3 serum concentrations in an ethnically diverse cohort of 53 RHD cases and 45 healthy controls from across Africa.

**Results:**

Ficolin-3 was found to be increased by 16% in RHD patients (*p* = 0.03) compared to controls, but polymorphisms did not associate with the risk of developing RHD nor with ficolin-3 concentrations. Carriers of the c.349del haploinsufficiency locus had normal levels of ficolin-3, while the previously described c.658 + 250 C > A RHD susceptibility locus was found equally in cases and controls. The higher serum ficolin-3 in RHD supports the potential role of this protein in RHD pathogenesis. However, these results suggest that rs532781899 and rs4494157 are not risk factors for the development of RHD in patients from sub-Saharan Africa and would not be reliable as early-stage markers of RHD susceptibility.

## Introduction

Rheumatic heart disease (RHD) is a long-term complication of rheumatic fever (RF) which is a sequel of recurrent *Streptococcus pyogenes* (group A *streptococcus*) infection [1]. While not completely understood, the pathobiology involves a complex autoimmune reaction, particularly in genetically susceptible individuals [2]. Characterised by chronic valvular lesions, RHD is the one of the most prevalent causes of acquired heart disease in children, adolescents, and young adults in lower-income countries [3].

Accurate diagnosis of RHD is a particularly critical issue, since early identification of RHD allows secondary prophylactic penicillin use, preventing recurrent exposure and further damage to heart valves [4]. However, in low-resourced settings, access to echocardiography, the gold standard for diagnosing RHD, is a challenge in terms of costs and technically competent staff. Furthermore, it relies on criteria that must balance sensitivity and specificity and, as such, invariably remains imperfect at diagnostic categorisation [5].

In ongoing efforts to identify clinical biomarkers, Salie et al. (2022) proposed a proteomic signature of severe RHD, developed using mass spectrometry, in an African cohort. Amongst the differentially expressed proteins, ficolin-3 was significantly reduced by 43% in cases compared to controls [6]. This reduction in serum ficolin-3 levels is hypothesised to stem from a prolonged inflammatory process following episodes of acute RF. Alternatively, it might be attributed to an inherent genetic predisposition leading to lower ficolin-3 levels [7].

Knowledge of the genetics of ficolin-3 is limited and conflicting. The intronic variant rs4494157 (c.658 + 250 C > A) was associated with increased ficolin-3 levels and susceptibility to both RF and RHD in an Egyptian cohort [7]. However, no genetic associations were found in a Brazilian study [8], where ficolin-3 was lower in RHD compared to controls. Furthermore, a frameshift deletion rs532781899 (c.349del) is a well characterised ficolin-3 deficiency locus– with a 50% reduction in ficolin-3 in European heterozygotes and complete deficiency in homozygotes [9–11]. Although identified in an immunodeficiency cohort [11], the role of this variant in RHD and other diseases is unclear [8].

Here, we investigated whether *FCN3* single-nucleotide polymorphisms (SNPs) are associated with ficolin-3 levels in African adolescents with RHD. We additionally sought to validate the in-silico findings that suggested a potential clinical utility for ficolin-3 as a biomarker for RHD.

Methods.

Participants with severe RHD were recruited, as previously described [12], from peri-urban settings across the African continent, along with ethnically and age-matched controls having no previous evidence of heart disease. Participants were eligible for inclusion in this study if they were between the ages of 10 and 23 years, and if both DNA and serum samples were available. Echocardiography was conducted to confirm the diagnosis of RHD.

Two *FCN3* SNPs were genotyped using Sanger sequencing: the *FCN3* deficiency variant rs532781899 (c.349del) in exon 5, and the reported RHD susceptibility locus rs4494157 (c.658 + 250 C > A) in intron 7. These variants of interest were amplified in each participant by polymerase chain reaction and subsequently sequenced using the BigDye™ Terminator v3.1 Cycle Sequencing Kit (Applied Biosystems™, USA). The Human Ficolin-3 ELISA kit was used to determine the circulating serum ficolin-3 concentration in the serum of participants. The assay was completed in duplicate and according to the manufacturer’s instructions (Assay Genie, Ireland).

Statistical analysis was performed using GraphPad Prism 10.0.2 (GraphPad Software, USA). Normality distribution of the variables was assessed using the Shapiro-Wilk test. Nonparametric Mann-Whitney and Kruskal-Wallis tests were used to compare differences in ficolin-3 concentration between the experimental groups. Differences between genotypes in cases and controls were measured using Chi-squared or Fisher exact tests, as appropriate. For all results, p-values less than 0.05 were considered significant.

## Results and discussion

**Fig. 1:**
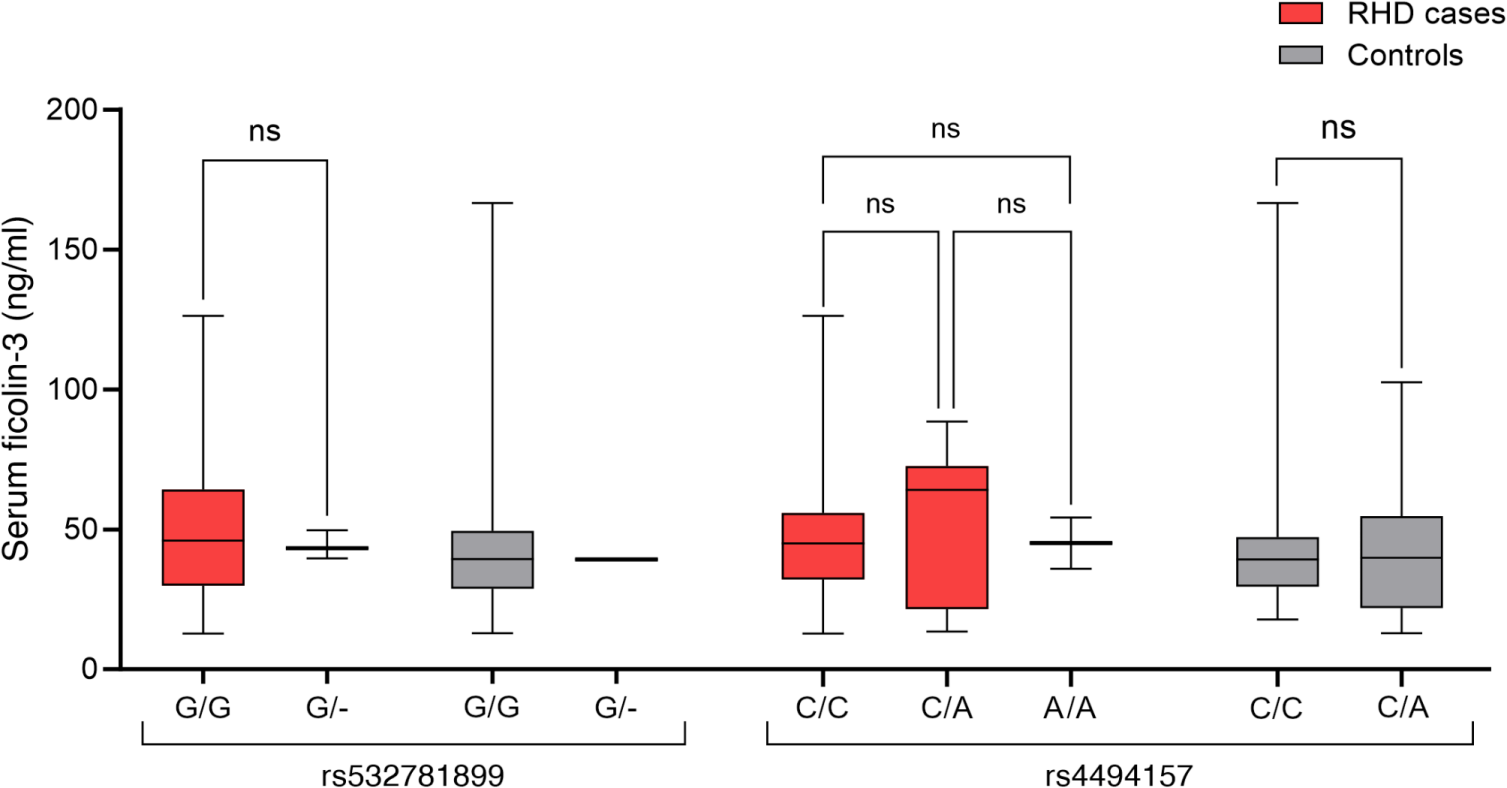
Distribution of serum ficolin-3 concentrations by genotype in cases and controls. The distributions of ficolin-3 concentrations were compared within genotypes using Mann-Whitney U tests. P-values < 0.05 was considered significant. *ns*,* not significant*

Fifty-three patients with severe RHD (age range: 10 to 23 years) and 45 healthy controls (age range: 16 to 23 years) from countries across Africa were included in this study (Table 1). Genotyping revealed that both SNPs were present in the study population and did not deviate from Hardy-Weinberg equilibrium (*p* > 0.05). Neither rs532781899 nor rs4494157 correlated with RHD disease status (Table 1) or serum ficolin-3 levels (Fig. 1).

**Table 1:** Characteristics and *FCN3* genotypes of the study population

	RHD (*n* = 53)	Controls (*n* = 45)	*P* value
**Median age**,** years (IQR)**	15.00 (12–20)	21.00 (18–22)	**< 0.0001**
**Female**,** n.** (%)	33 (62.26)	27 (60.00%)	0.8060
**Country of origin**,** n. (%)** Kenya Mozambique Namibia Nigeria South Africa Sudan Uganda Zambia Unknown	11 (20.75) 2 (3.77) 8 (15.09) 6 (11.32) 3 (5.66) 4 (7.55) 5 (9.43) 10 (18.86) 4 (7.55)	3 (6.67) 0 (0) 2 (4.44) 0 (0) 8 (17.78) 8 (17.78) 7 (15.56) 6 (13.33) 11 (24.44)	0.6489
**Median serum ficolin-3 concentration**,** ng/ml (IQR)**	45.90 (32.31)	39.25 (19.99)	**0.0302**
**rs532781899 genotype**,** n. (%)** G/G G/- -/-	50 (94.3) 3 (5.6) 0 (0)	44 (97.8) 1 (2.2) 0 (0)	0.6223
**rs4494157 genotype**,** n. (%)** C/C C/A A/A	42 (77.8) 9 (17.0) 2 (3.7)	37 (82.2) 8 (17.2) 0 (0)	0.6423
**rs4494157 allele frequency**,** n. (%)** C allele A allele	96 (87.3) 14 (12.7)	85 (90.4) 9 (9.6)	0.4795

*IQR*,* interquartile range; NA*,* not applicable; RHD*,* rheumatic heart disease*

The *FCN3* frameshift deletion rs532781899 was detected at similar proportions in RHD cases and healthy controls, indicating that it is unlikely to be a risk factor for the disease in our setting. This variant occurs in exon 5 of *FCN3*, where it causes premature termination of the coding transcript and a truncated ficolin-3 protein product (p.Leu117SerfsTer66) which lacks the fibrinogen-like domain. It is thought that this polymorphism disrupts the possibility for pathogen recognition [13]. In attempts to produce the recombinant protein with the alteration, the protein could not be expressed, suggesting that this variant leads to ficolin-3 deficiency [13]. Notably, four participants in this cohort were identified as carriers of the rs532781899 deletion and were not found to have any significant reduction in serum ficolin-3 concentration, contrary to the previously described effect of the polymorphism in heterozygous carriers [11, 13]. No homozygous carriers of this deletion were detected in the cohort.

The variant rs4494157 was detected in both patients and controls. This variant is located in intron 7 of *FCN3*, an intron that is thought to have characteristics of an enhancer region [14]; thus, variation in this region may affect gene expression through epigenetic modifications of CpG islands and histones. The A allele has been suggested to be a risk factor for the progression of RF to RHD [7]. Notably, the only two participants with the A/A genotype in our cohort were patients with severe RHD. However, this was statistically insignificant (*p* > 0.05). Thus, no valuable conclusions could be drawn regarding the association of this genotype with the disease state and its role as a potential risk factor.

The lack of an association between the genotypes of the investigated *FCN3* polymorphisms and RHD in this study suggests that these two SNPs (rs532781899 and rs4494157) do not influence susceptibility to RHD. Thus, our findings align more closely with what has been previously described in RF and RHD patients originating from Brazil, where no associations were found between the disease state and the genotypic distributions of the SNPs [8].

Ficolin-3 is the most abundant ficolin in sera and an integral component of the innate immune system, contributing to the host’s first line of defence against infections [15–17]. In the complement system, ficolin-3 forms complexes with mannose-binding lectin-associated serine proteases 1 and 2 to cleave complement components 4 and 2 to produce C3 convertase C4b2a, which thereby drives the activation of the pathway [16, 18]. This role in innate immunity, together with our previous identification of ficolin-3 as differentially expressed in RHD [6], makes Ficolin-3 an attractive potential biomarker of RHD. The serum ficolin-3 concentration was approximately 16% higher in RHD patients than in healthy controls in this study (Table 1).

While significant, this association is contrary to our prior in-silico predictions from the RHDGen study, which showed a reduction in ficolin-3 levels in patients with severe RHD compared to controls [6]. This may be explained by the larger cohort used in the in-silico analysis (215 patients and 230 controls), with no restriction on the age of participants. Thus, the higher ficolin-3 levels observed in our cohort could be attributed to the younger age of the participants with, possibly, earlier stages of disease progression. At this stage of the disease, maintained compensatory mechanisms may mitigate the effects of the hypothesised ficolin-3 consumption, which occurs during ongoing inflammation [19], while reduced ficolin-3 may be a characteristic of more severe forms of RHD.

## Limitations

The study was limited by the overall low number of participants and incomplete matching of the controls, although the sample size was calculated to give reasonable power (70%) to detect changes in ficolin-3 concentration based on the observed variability. Given the above-mentioned possibility that age may play a role in ficolin-3 levels, improved age-matching may be preferable in future studies to evaluate the role of ficolin-3 as a putative biomarker. As it has been previously reported that these variants are not in linkage disequilibrium [20], we did not consider the effects of variant combinations on disease susceptibility or ficolin-3 concentration. By focusing on two SNPs of interest, this study was unable to consider the role of other variants, or other genomic factors such as post-transcriptional and epigenetic events, in modulating gene expression. These two variants were selected because of their recent reported links to ficolin-3 deficiency and RHD; however, other common *FCN3* variants may have functional effects and should be considered. These include rs3813800 which has been associated with pulmonary infections in Chinese tuberculosis patients) [21], rs2504778 which has been associated with hypertension [22], and rs28362807 which formed part of a haplotype associated with elevated Ficolin-3 levels and susceptibility to leprosy [14]. Deeper, unbiased sequencing of *FCN3* is needed to elucidate the exact relationship between *FCN3* genetic variation, ficolin-3 concentration, and RHD pathogenesis.

## Conclusion

These results, although from a small sample, suggest that the *FCN3* polymorphisms rs532781899 and rs4494157 are not risk factors in the development of RHD in patients from Africa, and would not be reliable as early-stage markers of RHD susceptibility in an adolescent cohort. The higher serum ficolin-3 in RHD does support the potential role of this protein in RHD pathogenesis, although further research is required.

## Data Availability

The datasets generated and analysed in the current study are available in the University of Cape Town’s ZivaHub repository, 10.25375/uct.28547585.v1.

## Abbreviations

ELISA Enzyme: linked immunosorbent assay
RF: Rheumatic fever
RHD: Rheumatic heart disease
SNP: Single-nucleotide polymorphism
